# Maternal Thyroid Dysfunction and Gestational Anemia Risk: Meta-Analysis and New Data

**DOI:** 10.3389/fendo.2020.00201

**Published:** 2020-04-15

**Authors:** Yang Yang, Yuanyuan Hou, Huiru Wang, Xiaotong Gao, Xichang Wang, Jiashu Li, Weiping Teng, Zhongyan Shan

**Affiliations:** Department of Endocrinology and Metabolism, Institute of Endocrinology, The First Affiliated Hospital of China Medical University, China Medical University, Shenyang, China

**Keywords:** hypothyroidism, hyperthyroidism, TPOAb-positive, anemia, pregnancy

## Abstract

**Background:** Previous studies indicate the effects of thyroid dysfunction on adverse obstetric outcomes and fetal neurodevelopment, of which the results on gestational anemia are controversial. Here, we evaluated the influence of thyroid dysfunction on gestational anemia via published epidemiological articles and a new prospective study conducted by our team, respectively.

**Methods:** We searched studies on the PubMed, Embase, MEDLINE, and Cochrane databases as of November 2019, and conducted a prospective study in which participants underwent thyroid function and blood routine testing throughout pregnancy.

**Results:** The meta-analysis showed that pregnancies with overt hypothyroidism [OH; odds ratio (OR) = 3.74, 95% confidence interval (CI): 1.95–7.15] or that were thyroid peroxidase antibody (TPOAb)-positive (OR = 1.97, 95%CI: 1.19–3.26) had increased anemia risk, but similar results were not found in pregnancies with subclinical hypothyroidism (SCH) and hyperthyroidism. In the prospective study from our new data, the hypothyroid group had significant reductions in hemoglobin (Hb) (*P* = 0.048) and increased anemia risk (OR = 6.384, 95%CI: 2.498–16.311) during the second half of pregnancy. From the first to second half of pregnancy, the longitudinal reductions in Hb, erythrocyte (RBC), and hematocrit (Hct) levels were significantly increased in hypothyroid group.

**Conclusions:** Our meta-analysis indicates that untreated OH or TPOAb-positive pregnant women have increased risk of anemia. In addition, our new data showed that treated hypothyroidism is also a risk factor for anemia in the second half of pregnancy rather than in the first half. The results may guide strengthening of Hb monitoring in pregnancies with thyroid dysfunction.

## Introduction

Thyroid dysfunction is a common endocrine disease during pregnancy, and consists of overt hypothyroidism (OH), subclinical hypothyroidism (SCH), overt hyperthyroidism (OHyper), and thyroid peroxidase antibody (TPOAb)-positive status. In China, SCH prevalence in the first half of pregnancy is 5.96%, which is higher than that of OH (1). Thyroid dysfunction is associated with adverse pregnancy and neonatal outcomes, including placental abruption (2), preeclampsia (3), miscarriage (4, 5), gestational diabetes (6), neonatal death (7), intrauterine growth restriction (8) and neuropsychological development (9).

Anemia is a worldwide health problem affecting 33% of non-pregnant women and 38% of pregnant women (10). The prevalence of gestational anemia increases with the progress of pregnancy (11). The World Health Organization (WHO) divides anemia into normal cell, micro cell, and large cell anemia according to the form of the red blood cells (RBC). Iron deficiency anemia (IDA) accounts for 75% of anemia during pregnancy (12). Pregnant women are highly susceptible to IDA due to the increased demand for iron (13). Some studies have noted that anemia can weaken thyroid function by reducing TPO activity (14). Women with thyroid dysfunction were more likely to have anemia compared with euthyroid women (15). A cross-sectional study by Veltri et al. (16) showed that iron deficiency (ID) is related to high prevalence of thyroid autoimmune disease (TAI), higher serum thyroid-stimulating hormone (TSH), and lower free thyroxine (FT4) levels during the first trimester of pregnancy.

A clinical study that involved 15,000 pregnant women showed that SCH was not related to pregnancy anemia, whether treated or not (17). Sahu et al. (18) and Wang et al. (19) drew the same conclusion. However, Morchiladze et al. (20) and Hou et al. (21) drew the opposite conclusion. The impact of TPOAb on anemia is also controversial. In addition, the above studies are all cross-sectional studies. Therefore, we aimed to evaluate the relationship between thyroid dysfunction and anemia via meta-analysis and a longitudinal study.

## Materials and Methods

### Meta-Analysis

#### Literature Search Strategy

We searched for studies published on the PubMed, Embase, MEDLINE, and Cochrane databases by November 2019. The search terms used were: thyroid function, thyroid dysfunction, thyroid disease, hypothyroid, hypothyroidism, subclinical hypothyroid, subclinical hypo thyroidism, hyperthyroidism, hyperthyroid, subclinical hyperthyroid, subclinical hyperthyroidism, thyroid peroxidase antibody, anti-TPO, TPOAb, anemia, iron deficiency, Hb, hemoglobin, pregnancy, gestation.

#### Inclusion and Exclusion Criteria

Studies were considered eligible if they met the following criteria: investigated the relationship between gestational thyroid dysfunction and anemia; data on maternal thyroid dysfunction and gestational anemia could be extracted to analyze the 95% confidence interval (CI). Comments, conference abstracts, books, reports, and articles that could not be analyzed further were excluded. We also excluded studies that analyzed various types of thyroid dysfunction as a whole or studies that only compared Hb levels in patients with thyroid dysfunction. Studies involving subjects who were formerly diagnosed with other hematological disease and studies in which patients were treated with iron supplements were excluded from the study.

#### Data Extraction

Two authors carefully extracted the following information from each study: first author's name, publication year, pregnancy phase, country of population, diagnostic criteria for thyroid disease and anemia. In addition, we collected primary data on thyroid disease and anemia.

#### Quality Assessment of the Included Studies

We used the Newcastle-Ottawa scale (NOS) to assess the quality of the selected studies from three aspects: selection, comparability, and results, with a maximum of four, two, and three stars, respectively. Studies assigned >6 stars are considered high-quality studies (22).

#### Statistical Analysis

To assess the strength of the relationship between thyroid disease and anemia risk, we used Stata (version 15.1) to analyze the 95%CI to calculate the composite odds ratio (OR). The significance of the combined OR calculated using the Mantel–Haenszel statistical method was determined by the *Z*-test. *P* < 0.05 was considered significant. Heterogeneity was studied using the Cochrane Q test (*P* < 0.05 indicated statistical significance) and *I*^2^ statistic. *I*^2^ values of 25, 50, and 75% were used as evidence of low, moderate, or high heterogeneity, respectively. Random-effects model was used to pool the results when high heterogeneity was considered existed; fixed-effects model was used to pool the results if heterogeneity is low.

To assess the stability of the results, we performed a sensitivity analysis by omitting one report from each rotation and re-computing the pooled estimates of the remaining studies employing the metaninf command. Begg's test was conducted to assess publication bias.

### Prospective Study

#### Participants

The participants had been diagnosed with thyroid dysfunction from February 2016 to October 2019 at the First Affiliated Hospital of China Medical University endocrinology clinic before pregnancy and had been treated at our department until delivery. Women with history of other metabolic diseases, thyroid surgery, radioiodine treatment, or who had been treated with iron supplements before the first antenatal visit during pregnancy were excluded from the study. The control pregnant women were from the Subclinical Hypothyroidism Early Pregnancy (SHEP) project (23). The experimental procedure here has been approved by the China Medical University Ethics Committee and is consistent with the Helsinki Declaration. All participants signed written informed consent forms.

Up to October 2019, we enrolled a total of 198 pregnant women aged 23–45 years. During the first and second half of pregnancy, the thyroid function results and treatment in pregnant women with thyroid dysfunction were monitored. All participants completed questionnaires on history of thyroid disease, birth history, smoking, drinking, chronic diseases, and medication, and to monitor their Hb, RBC, and hematocrit (Hct) during the first and second half of pregnancy.

#### Diagnostic Criteria for Thyroid Dysfunction and Anemia

Serum TSH, FT4, free triiodothyronine (FT3), and TPOAb were measured using an electrochemiluminescence immunoassay on a Cobas Elecsys 601 unit (Roche Diagnostics, Basel, Switzerland).

For pregnancy, the TSH and FT4 reference intervals were 0.14–4.87 mIU/L and 12.35–20.71 pmol/L, respectively. For non-pregnant women, the TSH and FT4 reference intervals were 0.69–5.64 mIU/L and 12.27–19.10 pmol/L, respectively (24). TPOAb ≥ 50 IU/mL was considered positive (1). The diagnostic criteria for hyperthyroidism were decreased TSH levels combined with elevated FT4 levels. Hypothyroidism included OH with elevated TSH levels combined with decreased FT4 levels and SCH with elevated TSH levels with normal FT4 levels. According to the WHO guidelines, the lower threshold value for Hb in pregnancy is 110 g/L (25).

#### Statistical Analysis

Statistical analysis was performed using SPSS 25.0 (SPSS Inc., Chicago, IL, USA). Normally distributed data are expressed as the mean ± *SD*; non-normally distributed data are expressed as the median (5 and 95% interquartile range). We summarized demographic and laboratory characteristics as medians and interquartile ranges for continuous variables, or numbers and percentages for categorical variables. Normally and non-normally distributed count data were compared between groups using independent sample t-tests and the Mann-Whitney *U*-test, respectively. The chi-square test was used to test for categorical variables. The risk factors for anemia during pregnancy were assessed using logistic regression after adjusted for TPOAb, maternal age, body mass index (BMI), TSH, smoking and drinking, and *P* < 0.05 was considered statistically significant.

## Results

### Meta-Analysis of Thyroid Dysfunction in Gestational Anemia

#### Characteristics of the Included Studies

**Figure 1:**
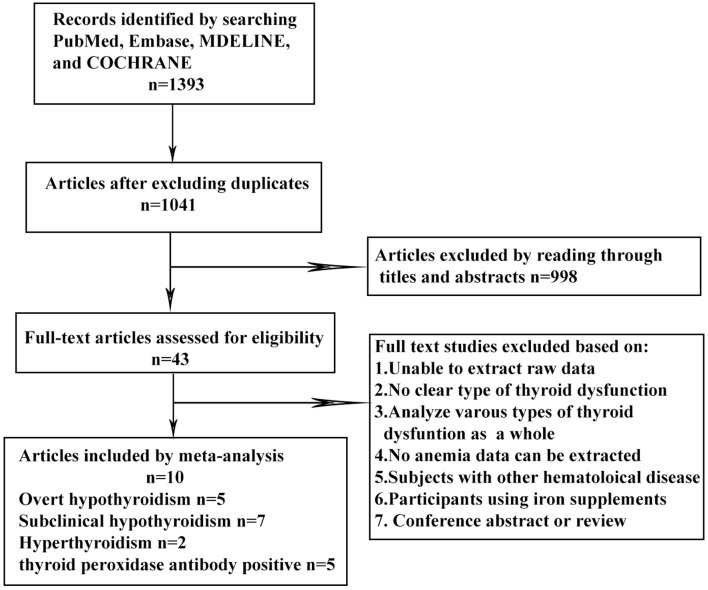
Flow chart of literature search and article selection.

The keyword search retrieved a total of 1,393 articles from the online databases. We excluded 998 articles by reading their titles and abstracts, and evaluated the remaining studies in their entirety, and 10 of them were included [(16–21, 23, 26–28); Figure 1]. According to the NOS, the included articles were high-quality articles. Table 1 shows the characteristics of all included articles.

**Table 1:** The characteristics of selected studies.

**Reference**	**Country**	**Items**	**Period**	**Measurement of thyroid function**	**Anemia diagnostic criteria**
Sahu et al. (18)	India	OH;SCH; hyperthyriodism	13–26 W	TSH: 0.5–5.5 mIU/L	Hemoglobin <110 g/L
Wang et al. (19)	China	SCH	Trimester I	FT4: 12–23.34 pmol/L	Hemoglobin <116 g/L
				TSH: 0.13–2.5 mIU/L	
Morchiladze et al. (20)	Georgia	OH	Trimester I	FT4: 10.3–24.5 pmol/L	Not mentioned
				TSH: 0.1–2.5 mIU/L	
				TPOAb+: >40 IU/L	
Li et al. (26)	China	OH; SCH; TPOAb+; hyperthyriodism	Trimester I	FT4: 11.49–18.84 pmol/L	Serum ferritin <20 μg/L
				TSH: 0.1–2.5 mIU/L	Hemoglobin <110 g/L
				TPOAb+: >5.61 IU/mL	
Veltri et al. (16)	Belgium	SCH; TPOAb+	Trimester I	FT4: 10.32–25.8 pmol/L	Serum ferritin <15 μg/L
				TSH: 0.3–4.0 mIU/L	
				TPOAb+:> 60IU/L	
				SCH: TSH > 2.5 mIU/L	
Hou et al. (21)	China	OH; SCH	10 W	FT4: 12–22.0 pmol/L	Not mentioned
				SCH:TSH>4.2 mU/L	
Yu et al. (23)	China	OH; SCH	Trimester I	FT4:12.35–20.71 pmol/L	Serum transferrin receptor > 4.4 mg/L
				TSH: 0.14–4.87 mIU/L	
Yang et al. (17)	China	SCH; TPOAb+	Trimester I	FT4: 12.91–22.35 pmol/L	Not mentioned
				TSH: 0.39–5.22 mIU/L	
				TPOAb+: >34 IU/L	
Meena (27)	India	TPOAb+	First half of pregnancy	TSH: 0.2–4.2 mIU/L	Not mentioned
Zhang et al. (28)	China	TPOAb+	Trimester I	TSH: 0.14–4.87 mIU/L	Serum ferritin <15 μg/L
				TPOAb+: >34 U/mL	

*OH, overt hypothyroidism; SCH, subclinical hypothyroidism; FT4, free thyroxine; FT3,free triiodothyronine; TSH, thyrotropin; TPOAb+, thyroid peroxidase antibody (TPOAb) positive; W, week*.

#### OH and Anemia

**Figure 2:**
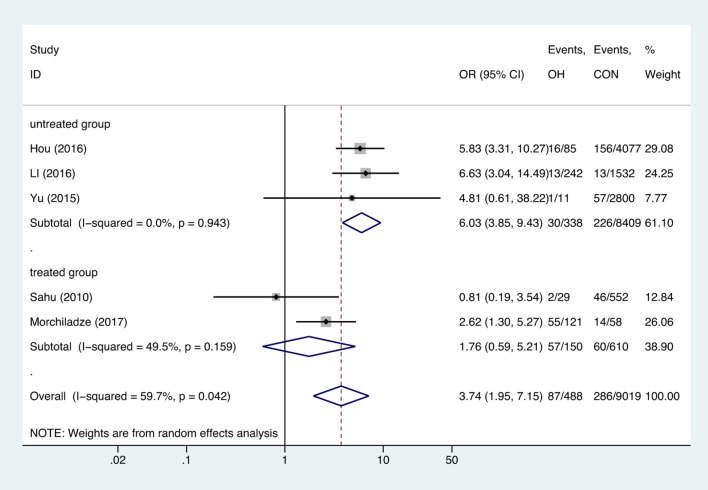
Forest plots of odds ratio and 95% confidence interval of pooled studies comparing pregnant women with overt hypothyroidism (OH) to euthyroid pregnant women (CON) for risk of gestational anemia.

Meta-analysis of the five studies that reported relevant data on the relationship between anemia and OH showed that the combined OR of anemia for OH pregnant women was 3.74 (95%CI: 1.95–7.15, *P* = 0, *I*^2^ = 59.7%), indicating that OH is associated with gestational anemia. Among the five articles, two did not mention antibodies and two identified type of anemia as IDA (18, 21, 23, 26). To explore the source of heterogeneity, we divided the five articles into two subgroups based on whether patients were treated. Subgroup meta-analysis indicated that untreated OH in pregnancy increased anemia risk (OR = 6.03, 95%CI: 3.85–9.43, *P* = 0, *I*^2^ = 0%), while treated OH in pregnancy was not associated with anemia (OR = 1.76, 95%CI: 0.59–5.21, *P* = 0.308, *I*^2^ = 49.5%). Heterogeneity was low following subgroup meta-analysis, indicating that the previous heterogeneity may have been due to different treatments (Figure 2). Begg's test (*P* = 0.462) did not indicate publication bias. Sensitivity analysis showed that the combined OR values of the remaining studies after one study had been removed remained stable.

#### SCH and Anemia

**Figure 3:**
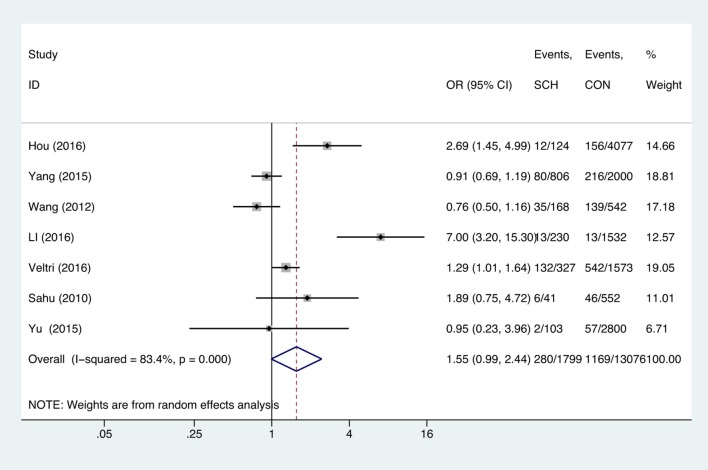
Forest plots of odds ratio and 95% confidence interval of pooled studies comparing pregnant women with subclinical hypothyroidism (SCH) to euthyroid pregnant women (CON) for risk of gestational anemia.

**Figure 4:**
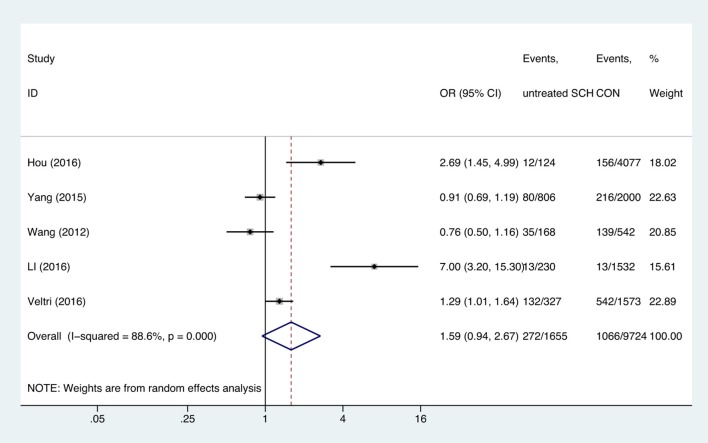
Forest plots of odds ratio and 95% confidence interval of pooled studies comparing untreated subclinical hypothyroid pregnant women (SCH) to euthyroid pregnant women (CON) for risk of gestational anemia.

Meta-analysis of the seven studies that reported relevant data on the association between anemia and SCH showed that SCH was not associated with anemia (OR = 1.55, 95%CI: 0.99–2.44, *P* = 0.056, *I*^2^ = 83.4%; Figure 3). Of these articles, there were five in which the patients were untreated. The combined results of the five studies found no significant association between untreated SCH with anemia (OR = 1.59, 95%CI: 0.94–2.67, *P* = 0.082, *I*^2^ = 88.6%; Figure 4). Among the seven included articles, three adopted the 2011 American Thyroid Association (ATA) guidelines as TSH > 2.5 mIU/L; the remaining four studies used pregnancy-specific reference ranges for diagnosing SCH. Subgroup analysis based on diagnostic criteria found that SCH remained unrelated to gestational anemia (Supplementary Figure 1). High heterogeneity persisted following the subgroup analysis, probably because the included articles had different diagnostic time of hypothyroidism and anemia. Begg's test (*P* = 0.548) did not indicate publication bias. Sensitivity analysis showed that the combined OR values of the remaining studies after one study had been removed remained stable.

#### Hyperthyroidism and Anemia

**Figure 5:**
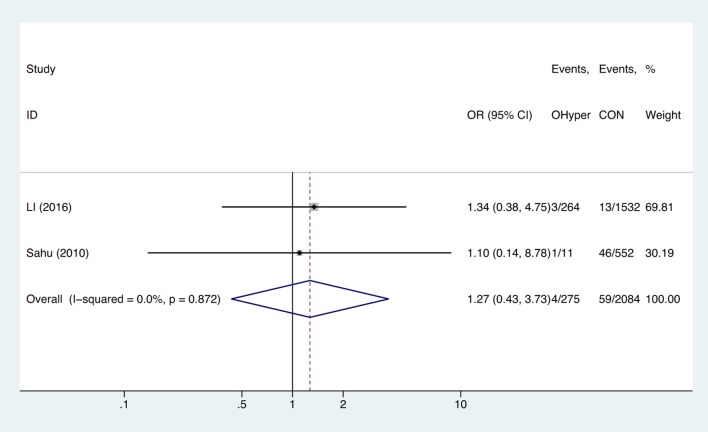
Forest plots of odds ratio and 95% confidence interval of pooled studies comparing pregnant women with hyperthyroidism (OHyper) to euthyroid pregnant women (CON) for risk of gestational anemia.

Two studies analyzed the effect of hyperthyroidism on gestational anemia. The combined OR of anemia for hyperthyroid pregnant women was 1.27 (95%CI: 0.43–3.73, *P* = 0.664, *I*^2^ = 0%), indicating that hyperthyroidism had no influence on gestational anemia (Figure 5).

#### TPOAb-Positive Status and Anemia

**Figure 6:**
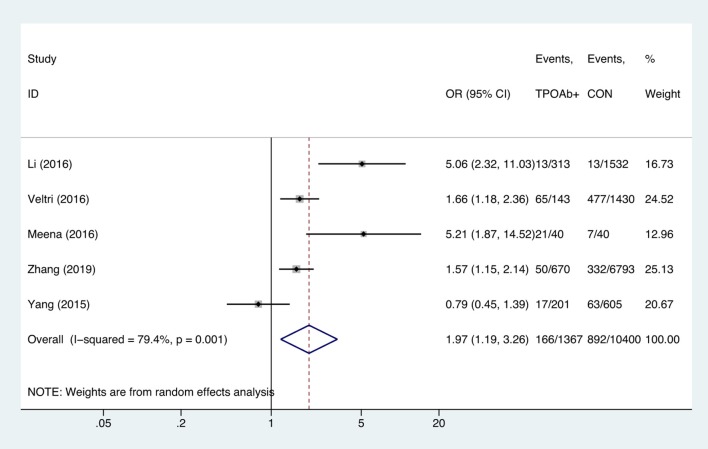
Forest plots of odds ratio and 95% confidence interval of pooled studies comparing thyroid peroxidase antibody-positive pregnant women (TPOAb+) to euthyroid pregnant women (CON) for risk of gestational anemia.

Compared with TPOAb-negative pregnant women, TPOAb-positive pregnant women had higher risk of anemia (OR = 1.97, 95%CI: 1.19–3.26, *P* = 0.009, *I*^2^ = 79.4%; Figure 6). Begg's test (*P* = 0.462) did not indicate publication bias, and sensitivity analysis showed that the combined OR values of the remaining studies after one study had been removed remained stable.

### Prospective Study

Table 2 shows the general characteristics and thyroid function of the included pregnant women. The age, body mass index (BMI), parity, and smoking and drinking status in the hypothyroid and hyperthyroid pregnant women were similar to that of euthyroid pregnant women. Hypothyroid pregnant women had higher FT4 levels after treatment (13.79 vs. 13.02 pmol/L, *P* < 0.05). In conservative observation or medication-treated hyperthyroid pregnant women, even though FT4, FT3, and TSH were in the normal range, the levels were nevertheless higher than that in euthyroid pregnant women (15.28 vs. 13.02 pmol/L, *P* < 0.001; 5.37 vs. 4.34 pmol/L, *P* < 0.001; 0.01 vs. 1.2 mIU/L, *P* < 0.001, respectively). The rates of TPOAb positive in both the hyperthyroid group and the hypothyroid group were higher than those in the euthyroid group.

**Table 2:** Demographic data and thyroid function test for thyroid dysfunction and euthyroid pregnant women.

	**Hypothyroidism**	**Hyperthyroidism**	**Euthyroid**
	***n* = 58**	***n* = 65**	***n* = 74**
**General characteristics**			
Age (y)^b^	31 (29-34)	30 (28-34)	30 (29-32)
BMI (kg/m2)^a^	21.75 ± 2.72	21.04 ± 2.07	21.72 ± 2.89
Nulliparity (%)	6.8 (4/59)	4.6 (3/65)	6.7 (5/74)
Smoking (%)	1.7 (1/59)	1.5 (1/65)	2.7 (2/74)
Drinking (%)	3.4 (2/59)	6.1 (4/65)	5.4 (4/74)
**Thyroid function test for first half of pregnancy**			
FT4 (pmol/L)^b^	13.79 (12.52–15.45)^c^	15.28 (13.81–17.96)^c^	13.02 (11.74–14.05)
FT3 (pmol/L)^b^	4.32 (4.0–4.63)	5.37 (4.6–6.81)^c^	4.34 (4.05–4.79)
TSH (mIU/L)^b^	1.33 (0.63–2.31)	0.01 (0–−0.38)^c^	1.20 (0.82–1.96)
TPOAb positive (%)	46.6 (27/58)^c^	55.4 (36/65)^c^	28.4 (21/74)

*BMI, body mass index;FT4, free thyroxine; FT3,free triiodothyronine; TSH, thyrotropin; TPOAb, thyroid peroxidase antibody*.

a *Means ± SD*,

b *Median (interquartile range)*,

c *P < 0.05 compared with euthyroid group*.

Compared with euthyroid pregnant women, there were no significant differences in Hb, RBC, and Hct levels in hypothyroid pregnant women during the first half of pregnancy. In the second half of pregnancy, Hb levels were significantly reduced (115 vs. 120 g/L, *P* = 0.048) and the prevalence of anemia was increased (OR = 6.384, 95%CI: 2.498–16.311) in the hypothyroid pregnant women after adjusted for TPOAb, maternal age, body mass index (BMI),nulliparity, smoking and drinking (Tables 3, 4). RBC levels were significantly higher in hyperthyroid pregnant women throughout pregnancy, while other hematological parameters showed no differences (Table 3).

**Table 3:** Comparison of hematological parameters in thyroid dysfunction and euthyroid pregnant women.

	**Hypothyroidism**	**Hyperthyroidism**	**Euthyriod**
**First half of pregnancy**			
*N*	58	65	74
Gestational weeks (w)^c^	12.5 (10-16)	13 (8-16)^d^	14 (10.75–17)
Hb (g/L)^c^	127.5 (118.75–132.25)	129 (123–135.5)	125.5 (118.5–133)
RBC (10^12^/L)^c^	4.29 (3.89–4.60)	4.40 (4.16–4.59)^d^	4.16 (3.83–4.37)
HCT (L/L)^a^	0.38 ± 0.04	0.38 ± 0.03^b^	0.37 ± 0.03
**Second half of pregnancy**			
*n*	52	61	71
Gestational weeks (w)^c^	27 (24-31)^d^	29 (25.5–32)	29 (25-33)
Hb (g/L)^c^	115 (108–123)^d^	120 (116–127)	120 (114–123)
RBC (10^12^/L)^c^	3.82 (3.59–4.17)	4.01 (3.73–4.3)^d^	3.78 (3.61–3.99)
HCT (L/L)^a^	0.35 ± 0.03	0.36 ± 0.03	0.35 ± 0.03
**Change from first half to second half of pregnancy**			
Hb (g/L)^c^	12.60 ± 9.34^b^	8.46 ± 8.36	7.85 ± 9.23
RBC (10^12^/L)^c^	0.52 ± 0.40^b^	0.41 ± 0.35	0.32 ± 0.36
HCT (L/L)^a^	0.03 (0.01–0.05)^d^	0.02 (0–0.04)	0.01 (0–0.03)

*Hb, Hemoglobin; RBC, red blood cell; HCT, hematocrit; n, the number of patients*.

a *Means ± SD*,

b *P < 0.05 compared with euthyroid group*,

c *Median (interquartile range)*,

d *P < 0.05 compared with euthyroid group*.

**Table 4:** Logistic regression evaluating the risk factors for gestational anemia.

	**Anemia (first half of pregnancy)**		**Anemia (second half of pregnancy)**	
	**OR (95%CI)**	***P***	**OR (95%CI)**	***P***
Hypothyroid pregnant women	1.871 (0.403–8.677)	0.424	6.384 (2.498–16.311)	0
Hyperthyroid pregnant women	1.306 (0.266–6.425)	0.742	1.447 (0.515–4.069)	0.484

*Multivariable model adjusted for TPOAb, maternal age, body mass index (BMI), nulliparity, smoking and drinking. CI, confidence interval*.

Compared with euthyroid pregnant women longitudinal reductions in the Hb were significantly in increased (12.60 ± 9.34 g/L vs. 7.85 ± 9.23, *P* = 0.006) in hypothyroid pregnant women from the first to second half of pregnancy. In addition, longitudinal reductions in the RBC and Hct levels were also increased (0.52 ± 0.40 vs. 0.32±0.36 10^12^/L, *P* = 0.01; 0.03 vs. 0.01 L/L, *P* = 0.015) in pregnant women with hypothyroidism. (Table 3).

## Discussion

The present article assessed the relationship between maternal thyroid dysfunction and gestational anemia. The meta-analysis showed that OH and TPOAb-positive status were risk factors of gestational anemia, while SCH and hyperthyroidism were not. In new data from our team, we found that Hb levels of pregnant women with hypothyroidism were lower during the second half of pregnancy and longitudinal reductions in the Hb, RBC, and Hct levels were higher from the first to second half of pregnancy. Moreover, RBC levels were significantly increased in the hyperthyroid pregnant women throughout pregnancy.

Affected by factors such as human chorionic gonadotropin (hCG), iodine level, and serum thyroxine-binding globulin (TBG), thyroid function levels fluctuate during pregnancy (29). This means that the diagnostic criteria for thyroid dysfunction during pregnancy differ from those in non-pregnancy. At present, several studies have noted that abnormal thyroid function during pregnancy can lead to obstetric adverse outcomes and adverse fetal neurodevelopment, including miscarriage, gestational diabetes mellitus, preeclampsia, and intrauterine growth restriction (IUGR) (30, 31). Therefore, it is necessary to assess thyroid function during pregnancy based on specific reference values for people with abnormal thyroid function.

Due to the increased demand for nutrition during pregnancy and the plasma volume increasing more quickly than red cell mass, anemia is prone to occur in middle and late pregnancy, especially IDA. A few studies have reported that ID can affect thyroid function (32, 33). Iron deficiency without anemia (ID – A) and severe iron deficiency with anemia (ID + A) rat models showed similar results that both of them could cause serum TT4 decreased as well as TPO activities reduction during pregnancy (34). However, the mechanism between anemia and thyroid function remains unclear; a few studies have shown that the mechanism by which ID affects thyroid function is likely to impair the efficacy of iodized salt and TPO activity as well as the conversion of T4 to T3 (14, 35, 36). A meta-analysis reported that when no consideration age and gender restrictions, the risk of anemia was increased in patients with OH and SCH compared with euthyroid participants. While prevalence of anemia increases with age. When conducting a subgroup analysis of age, the study found that the pooled OR for the SCH younger than 50 years old was 1.15 (0.77–1.74) indicating no relationship between anemia and SCH younger than 50 years old (15). However, no meta-analysis had been conducted to investigate the effect of maternal thyroid dysfunction on anemia. A cross-sectional study in China indicated that pregnancies with IDA show lower FT4 levels compared with the control group during the first trimester of pregnancy (37). Another article showed that Hb correlated positively with FT4 and FT3 and negatively with TSH (5).

OH is characterized by elevated TSH concentrations with low FT4 and FT3 concentrations. As OH has a definite adverse effect on obstetric and child development outcomes during pregnancy, the 2017 ATA guidelines recommend that OH be treated as early as possible (38). In the present meta-analysis, we found that the OR for anemia in treated OH pregnant women was 1.76 (95%CI: 0.59–5.21, *P* = 0.308), indicating that treatment can eliminate the effects of OH on anemia. The results prompt the diagnosis and treating of gestational OH as soon as possible.

SCH is a condition with elevated TSH concentrations. The 2011 ATA guidelines recommend using fixed upper limits of 2.5 or 3.0 mIU/L for the first and second or third trimesters, respectively (39). The present meta-analysis indicates that SCH is not associated with gestational anemia. Of seven included studies, three adopted the diagnostic criteria of the 2011 guidelines, but the present analysis suggests that, with either criteria, SCH is not a risk factor for anemia during pregnancy.

Currently, there only two studies analyzed the effect of hyperthyroidism on gestational anemia. One of them is a prospective study conducted by Sahu et al. (18). The article showed that both overt and subclinical hyperthyroidism had no influence on gestational anemia. And another article came to similar results (26). Nevertheless, more large-sample epidemiological studies are needed to clarify this issue.

Euthyroid TAI is easily overlooked because of the lack of specific clinical symptoms. It had also been proposed that positive TPOAb can adversely affect pregnancy and neonatal outcomes (9, 40, 41). Here, we focused the effect of TPOAb on gestational anemia and found increased risk of gestational anemia in TPOAb-positive pregnant women. On one hand, the probable reason is that TPO is a heme-dependent protein, which interacts with iron levels (14). On the other hand, it may be due to the effect of inflammatory mediators on erythropoiesis, similar to other autoimmune diseases (42).

In order to better explain the relationship between thyroid dysfunction and gestational anemia, we conducted a prospective study and observed that Hb levels decreased significantly in hypothyroid pregnant women, and precisely show that hypothyroidism is an independent risk factor for anemia in the second half of pregnancy rather than in the entire pregnancy. From the first to second half of pregnancy, there was increased Hb, RBC, and Hct reduction in the hypothyroid pregnant women. Compared with the results of our meta-analysis, the longitudinal study showed that treated hypothyroidism is also a risk factor for gestational anemia in the second half of pregnancy. The possible reasons are: (1) Anemia was diagnosed at different gestational periods. Two articles included in the meta-analysis on treated hypothyroidism did not mention the exact timing of the anemia diagnosis. However, our prospective study highlights the impact of treated hypothyroidism on anemia risk in the second half of pregnancy; (2) The course of hypothyroidism is different. Most of the patients included in the meta-analysis were new-onset hypothyroidism in the first trimester, while the patients in the prospective study had a longer course of hypothyroidism.

To our knowledge, this is the first meta-analysis and longitudinal study focusing on the influence of abnormal thyroid function on anemia during pregnancy. There remain some limitations to this study. Although positive TPOAb status had an effect on anemia, we did not consider antibody factors for the patients with hypothyroidism included in our meta-analysis and prospective study. Due to the influence of the region and the publication year, the diagnostic criteria for SCH were not consistent. Although we had performed subgroup analyses according to diagnostic criteria, the effects cannot be completely eliminated. We included 10 related articles in the meta-analysis, most of which came from Asia. Due to the small number of articles on the effects of thyroid dysfunction on gestational anemia, there were only two articles included in our subgroup analysis of treated or untreated overt hypothyroidism. In addition, the number of patients included in our longitudinal study was small. It is necessary to expand the population for further exploration.

In summary, untreated OH and positive TPOAb status are associated with increased anemia risk. Treated hypothyroidism is a risk factor for anemia in the second half of pregnancy rather than in the first half. The results may guide strengthening of Hb monitoring in pregnant women with thyroid dysfunction and guide the timely treatment of pregnant women with anemia.

## Data Availability Statement

All datasets generated for this study are included in the article/Supplementary Material.

## Ethics Statement

The studies involving human participants were reviewed and approved by China Medical University Ethics Committee. The patients/participants provided their written informed consent to participate in this study.

## Author Contributions

ZS conceived the study, interpreted the data, and critically revised the report. WT critically revised the report. YY searched, collected, analyzed, and interpreted the data, and drafted and critically revised the report. YH, HW, XG, XW, and JL searched and collected data.

### Conflict of Interest

The authors declare that the research was conducted in the absence of any commercial or financial relationships that could be construed as a potential conflict of interest.

## Supplementary Material

Supplementary Figure 1 Forest plots of odds ratio and 95% confidence interval of pooled studies comparing pregnant women with subclinical hypothyroidism (SCH) to euthyroid pregnant women (CON) for risk of gestational anemia based on different diagnostic criteria.
