# Supplementary figures and images for: Maternal Thyroid Dysfunction and Gestational Anemia Risk: Meta-Analysis and New Data
Source: Front Endocrinol (Lausanne). 2020 Apr 15;11:201. doi: 10.3389/fendo.2020.00201 (PMC7174567; doi:10.3389/fendo.2020.00201)

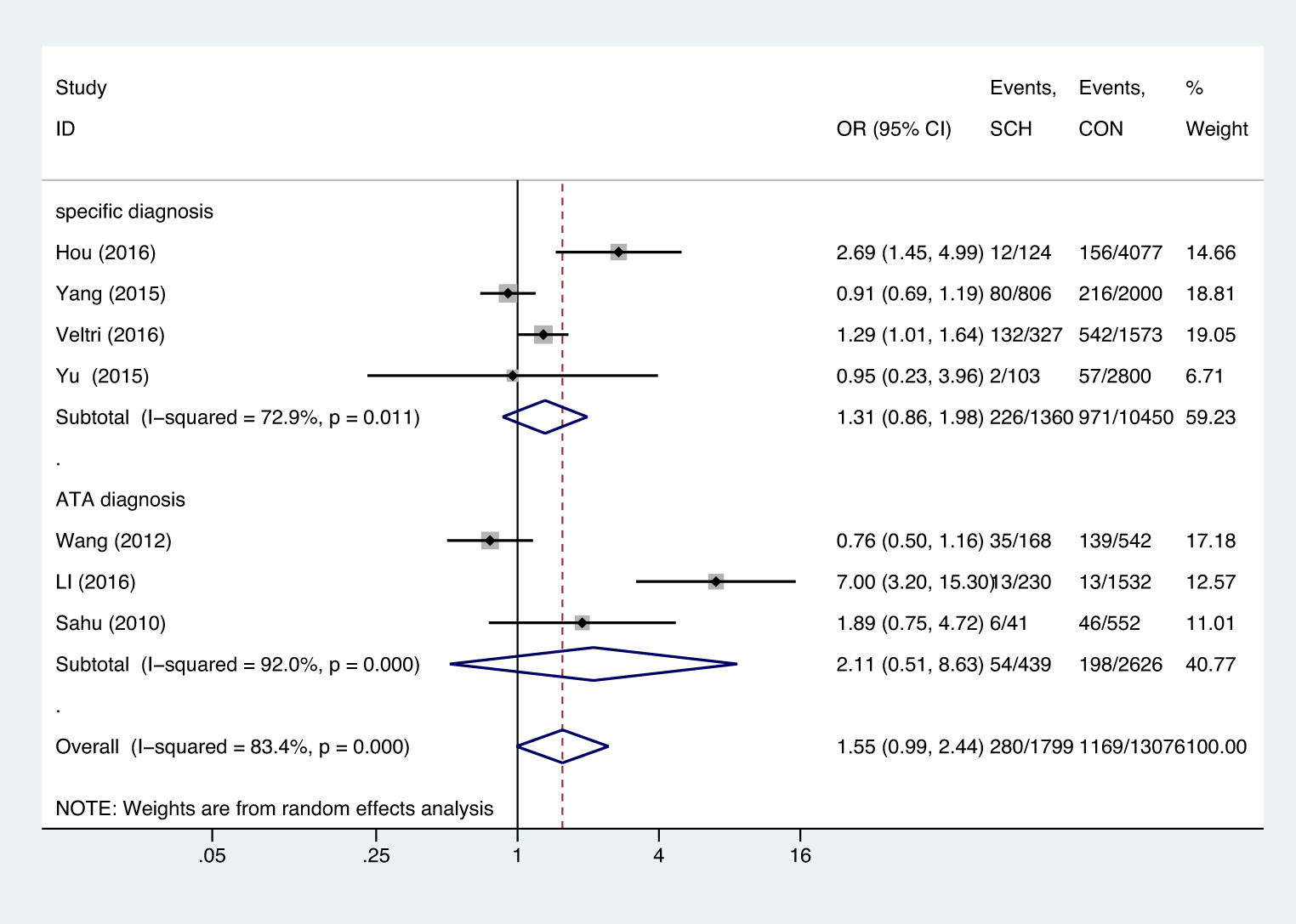

Supplement: Supplementary Figure 1 — Forest plots of odds ratio and 95% confidence interval of pooled studies comparing pregnant women with subclinical hypothyroidism (SCH) to euthyroid pregnant women (CON) for risk of gestational anemia based on different diagnostic criteria. [file Image_1.JPEG]
